# Soluble HLA-G and HLA-G Bearing Extracellular Vesicles Affect ILT-2 Positive and ILT-2 Negative CD8 T Cells Complementary

**DOI:** 10.3389/fimmu.2020.02046

**Published:** 2020-08-21

**Authors:** Esther Schwich, Gia-Gia T. Hò, Joel LeMaoult, Christina Bade-Döding, Edgardo D. Carosella, Peter A. Horn, Vera Rebmann

**Affiliations:** ^1^Institute for Transfusion Medicine, University Hospital Essen, University Duisburg-Essen, Essen, Germany; ^2^Institute for Transfusion Medicine, Hannover Medical School, Hanover, Germany; ^3^Commissariat à l’Energie Atomique et aux Energies Alternatives (CEA), Direction de La Recherche Fondamentale (DRF), Service de Recherche en Hémato-Immunologie (SRHI), Hôpital Saint-Louis, Paris, France; ^4^Institut de Recherche Saint-Louis, Université de paris, Paris, France

**Keywords:** HLA-G, ILT-2, immune checkpoint, extracellular vesicles, exosomes, breast cancer

## Abstract

Tumor immune escape is associated with both, the expression of immune checkpoint molecules on peripheral immune cells and soluble forms of the human leukocyte antigen-G (HLA-G) in the blood, which are consequently discussed as clinical biomarker for disease status and outcome of cancer patients. HLA-G preferentially interacts with the inhibitory receptor immunoglobulin-like transcript (ILT) receptor-2 in the blood and can be secreted as free soluble molecules (sHLA-G) or via extracellular vesicles (EV). To investigate the contribution of these two forms to the expression of checkpoint molecules in peripheral blood, we primed peripheral blood mononuclear cells with purified soluble sHLA-G1 protein, or EV preparations derived from SUM149 cells transfected with membrane-bound HLA-G1 or control vector prior to anti-CD3/CD28 T cell activation. Our study demonstrated that priming of PBMC with sHLA-G1 protein prior to 48 h activation resulted in enhanced frequencies of ILT-2 expressing CD8^+^ T cells, and in an upregulation of immune checkpoint molecules CTLA-4, PD-1, TIM-3, and CD95 exclusively on ILT-2 positive CD8^+^ T cells. In contrast, when PBMC were primed with EV (containing HLA-G1 or not) upregulation of CTLA-4, PD-1, TIM-3, and CD95 occurred exclusively on ILT-2 negative CD8^+^ T cells. Taken together, our data suggest that priming with sHLA-G forms induces a pronounced immunosuppressive/exhausted phenotype and that priming with sHLA-G1 protein or EV derived from HLA-G1 positive or negative SUM149 cells affects CD8^+^ T cells complementary by targeting either the ILT-2 positive or negative subpopulation, respectively, after T cell activation.

## Introduction

The human leukocyte antigen-G (HLA-G) belongs to the non-classical class I HLA molecules and can exist in different isoforms expressed either as membrane-anchored structures or as secreted molecules (1–4). Additionally, HLA-G can be released as membrane-anchored molecules from various cell types via extracellular vesicles (EV) (5). EV are phospholipid bilayer-enclosed vesicles that are present in biofluids and cell culture media (6). Assembly of EV depends on their cell of origin and differs remarkably encompassing a broad spectrum of antigens, cell surface-expressed receptors and/or ligands, metabolites, and nucleic acids (7). Generally, the unique molecular signature of EV guides their biodistribution, uptake and internalization (7). As multifactorial vehicles, EV orchestrate various systemic processes, triggering changes of the state of the recipient cell (8). In malignancies, EV play a critical role in the establishment and maintenance of the tumor microenvironment (TME) (6), which enables tumor development by continuous crosstalk between tumor cells and their microenvironment and by providing the tumor with cellular and soluble components including nutrients, oxygen, metabolites, and several other soluble factors (9). EV can either directly fuse with a target cell enabling the transfer of bioactive molecules to both, adjacent and distant sites, or be internalized via phagocytosis, endocytosis or micropinocytosis, thereby contributing to an intracellular signaling mechanism (10). Of note, fusion depends on an acidic micro-environment which naturally occurs inside tumors (11–14), while uptake and internalization of EV are primarily receptor-mediated via adhesion molecules (15). Thereby, tumor-derived EV (TEV) may represent an alternative mechanism of immunosurveillance deficiency impairing diverse immune cell lineages (6).

HLA-G preferentially serves as a ligand for inhibitory receptors present on different immune cells including the immunoglobulin-like transcript (ILT) receptor-2 (LILRB1/CD85j), ILT-4 (LILRB2/CD85d) and the killer immunoglobulin-like receptor 2DL4 (KIR2DL4/CD158d). ILT-2 is broadly expressed on monocytes, B cells, dendritic cells, and a subset of natural killer (NK) and T cells, whereas ILT-4 expression is myeloid-specific (16). Thus, HLA-G is able to impair functions of effector cells of both, the adaptive and the innate immune system. The ILT-2 receptor interacts with HLA-G molecules associated to β2-microglobulin and HLA-G dimers bind to ILT-2 with a higher affinity and avidity than monomers (17). Of note, similar to classical soluble HLA class I, soluble HLA-G (sHLA-G) can interact with the CD8 T cell co-receptor, which increases surface expression and secretion of FasL – the ligand of the Fas (CD95) receptor – inducing cell apoptosis (18).

Physiologically, HLA-G has a restricted tissue expression, whereas neo-expression of HLA-G and its diverse structures is induced in various pathological situations (2). Due to the role of HLA-G in tumor immune escape, it is proposed to be an immune checkpoint (IC) molecule (19). Indeed, expression of HLA-G or sHLA-G has been associated with poor survival, prognosis, therapy response, clinical status, and outcome in various malignancies [reviewed in Carosella et al. (19)]. Lately, HLA-G bearing EV (HLA-G_EV_) originated from liquid biopsies of blood samples derived from breast and ovarian cancer patients have been introduced as novel cancer biomarker (20, 21). Strikingly, in these studies exclusively HLA-G_EV_, but not sHLA-G, were of prognostic relevance suggesting self-contained effects of both structures. However, the structural diversity concerning monomers, dimers, and HLA-G expressing EV in liquid biopsies such as peripheral blood samples makes it difficult to implement HLA-G as a meaningful clinical biomarker with its functional consequences for peripheral immune effector cells (22). In this context, it is of note that the ILT-2 receptor is the sole inhibitory HLA-G receptor being expressed on peripheral blood cells, albeit only a minority of blood effector cells express ILT-2 (23). Thus, it has been proposed that the functional consequences of HLA-G and its soluble forms for immune cells in the blood should be focused on HLA-G sensitive effectors, namely the ILT-2 positive ones (23).

Besides HLA-G and ILT-2, additional IC molecules such as programmed cell death protein-1 (PD-1), cytotoxic T-lymphocyte-associated protein (CTLA-4), T-cell immunoglobulin and mucin-domain containing-3 (TIM-3), and CD95 are associated in tumor-driven immune escape mechanisms acting locally at the tumor site or systemically in the peripheral blood (24–26). The continuous up-regulation and co-expression of multiple IC, being often observed in cancer and chronic infections, are indicative for an immunosuppressive/exhausted phenotype of T cells and are associated with loss of effector functions and immunosurveillance (27, 28). Hitherto, no data exists on the relation between sHLA-G or HLA-G_EV_ and the expression of ICs on peripheral blood cells. Hence, the aim of this study was to analyze the contribution of soluble forms of HLA-G to the surface expression of IC molecules. Purified sHLA-G1 molecules (29) and EV preparations derived from the human breast cancer (BC) cell line SUM149 either stable transfected with HLA-G1 or with a control vector served as antigen sources in functional assays. To model whether presence of sHLA-G1 or HLA-G_EV_ in the peripheral blood modulates immune effector cells regarding their expression of ICs, peripheral blood mononuclear cells (PBMC) were primed with sHLA-G1 or with HLA-G1 positive or negative EV preparations overnight prior to T cell activation with anti-CD3/CD28. As EV harbor multiple types of molecules, structures, and genetic information, we placed emphasis on both, the ILT-2 positive and ILT-2 negative T cell population.

## Materials and Methods

### Cell Culture

Human BC cell line SUM149 was stable transfected with a GFP construct targeting HLA-G G1 (SUM149 LV2 G1-GFP) or with a control vector encoding GFP only (SUM149 LV2 N3-GFP). Cells were cultured in RPMI-1640 supplemented with 1% Penicillin/Streptomycin (both Thermo Fisher Scientific, Darmstadt, Germany) and 10% FBS Good Forte (PAN-Biotech GmbH, Aidenbach, Germany) at 37°C and 5% CO_2_. Conditioned media (CM) were collected for EV enrichment and frozen at −20°C.

### Isolation and Characterization of Extracellular Vesicles Derived From Conditioned Media

To isolate EV derived from CM of HLA-G1 transfected SUM149 cells (G1 EV) and the respective control cells (N3 EV), CM were thawed and centrifuged at 2,800 × *g* for 30 min at 4°C and concentrated by tangential flow filtration (Repligen, Breda, Netherlands) with a 750 kDa/115 cm^2^ mPES filter (D02-E500-05-N). The concentrate was subjected to ultra-centrifugation at 100,000 × *g* for 2 h at 4°C in a swinging bucket SW40 Ti rotor (Beckman Coulter, Krefeld, Germany). The pelletized EV were resuspended in 0.9% NaCl supplemented with 1% Penicillin/Streptomycin (Thermo Fisher Scientific).

EV fractions were analyzed as previously recommended as a minimal requirement for the definition of EV (30, 31). Nanoparticle tracking analysis (NTA) on the ZetaView Laser Scattering Video Microscope (Particle Metrix, Meerbusch, Germany) and its corresponding software (version 8.03.08.02) revealed a size distribution (mean ± SD nm) of 136.7 ± 3.3 and 133.4 ± 3.3 for the G1 EV or N3 EV preparation (Supplementary Table S1), which corresponds to the known size of EV, ranging between 30 and 150 nm (32). Particle concentration was determined by NTA and protein concentration was defined by protein assay (Thermo Fisher Scientific) (Supplementary Table S1). Expression of components associated with EVs and classical HLA class I was verified by SDS PAGE and western blot (Supplementary Figure S1A). 15 μg of EV suspensions were used for immunoblotting and 10 μg cell lysate derived from the respective cells served as control. Both preparations showed the typical EV marker profile including presence of TSG101 (clone: T5701; Sigma-Aldrich, St. Louis, MO, United States), classical HLA class I [α-heavy chain HLA class I; (33)], Syntenin (clone EPR8102; Abcam), and CD81 (clone: 5A6; BioLegend, Koblenz, Germany) and absence of Calnexin (Abcam) excluding cellular protein contamination. Additionally, western blot analysis revealed that HLA-G (clone 4H84; Exbio, Praha, Czechia) present in both, cell lysate and EV fraction of SUM149 LV2 G1-GFP cells and absent in cell lysate and EV fraction of the control SUM149 LV2 N3-GFP cells (Supplementary Figure S1B). Both, the NTA results and the EV marker profile fulfill the minimal requirement for the definition of EV (30, 31).

### Stimulation of Peripheral Blood Mononuclear Cells

Frozen PBMC of healthy donors [for isolation and storage of PBMC see Kordelas et al. (34)] were thawed in complete medium consisting of RPMI-1640, 1% Penicillin/Streptomycin, 10% human AB serum (Transfusion Medicine, University Hospital Essen, Germany), and 0.556 μg DNAse (Roche, Mannheim, Germany). In a 96-U-bottom plate 6 × 10^5^ PBMC/well were cultured in 200 μL of DNAse-free complete medium at 37°C and 5% CO_2_ alone (control), in the presence of 1.2 ng purified HLA-G1 (sHLA-G1) protein (29) or in presence of 40 μg EV either derived from HLA-G1 transfected SUM149 cells (G1 EV) or from the respective control cells (N3 EV). 40 μg G1 EV corresponds to a mount of 1.2 ng HLA-G1 defined by HLA-G ELISA as previously described (20, 21, 35, 36). After 24 h, primed and unprimed PBMC were stimulated with beads coated with CD3/CD28 (Thermo Fisher Scientific) in a bead to cell ratio of 1:3 for 48 h. Influence of stimulation on the expression of IC molecules on T cells was assessed (Supplementary Figure S2). Additionally, viability of T cells upon stimulation and priming was analyzed (Supplementary Figure S3).

### Flow Cytometric Analysis

LIVE/DEAD Violet^TM^ Dead Cell Stain Kit was used according to manufacturer’s instructions (Thermo Fisher Scientific) to analyze cell viability. Surface expression was analyzed by staining with fluorchromes-conjugated mononuclear antibodies targeting CD3 (BV510 clone OKT3), CD8 (PerCP-Cy5.5 clone SK1), PD-1 (AF488 clone EH12.2H7), CD95 (BV510 clone DX2), TIM-3 (PerCP/Cy5.5 clone F38-2E2), or CTLA-4 (BV605 clone BNI3). All antibodies were provided by BioLegend (Koblenz, Germany) with the exception of CD3 (Beckman Coulter). Isotype matched antibodies served as negative controls (BD Bioscience, Heidelberg, Germany). Samples were subjected to multicolor flow cytometry using a CytoFlexS cytometer (Beckman Coulter). Data acquisition of at least 200.000 events was performed with CytExpert Version 2.1 software (Beckman Coulter) and analyzed with Kaluza Analysis 2.1 software. General gating strategy for flow cytometric analysis is visualized in Supplementary Figure S4. Analysis strategy for multiple-positive T cells is given in Supplementary Figure S5.

### Statistical Analysis

Data is presented as median with the 10th and 90th percentile. Frequencies of a certain cell population are either expressed as% or as fold change (FC). For FC, frequencies of sHLA-G1- or EV-primed cells were normalized to the corresponding stimulations obtained without priming. After testing for Gaussian distribution, statistical significance was determined by paired *t*-tests or Wilcoxon test for testing of two groups or by two-way ANOVA for comparison of multiple groups. Statistical analysis was performed by using GraphPad Prism V8.3 software (GraphPad Software, San Diego, CA, United States). *p*-values <0.05 were considered to be statistically significant.

## Results

### Priming With sHLA-G1 Modulates the ILT-2 Expression of CD8^+^ T Cells

To mimic whether the expression of ICs on T cells can be modulated by the presence of sHLA-G1 in the peripheral blood, PBMC (*n* = 6) of healthy individuals were primed with sHLA-G1 overnight prior to stimulation with anti-CD3/CD28. Flow cytometric analysis (Supplementary Figure S2) revealed similar frequencies of ILT-2 positive CD4^+^ and CD8^+^ T cells [median (range) in%: 19.6 (14.8–24.5) and 22.7 (11.5–39.6), respectively] in unprimed PBMC upon stimulation with CD3/CD28. However, priming with sHLA-G1 resulted in a significant increase of ILT-2 on the CD8^+^ T cell subpopulation [53.8 (22.2–64.9)], while ILT-2 on CD4^+^ T cells was only marginally increased [23.8 (14.4–41.8); Supplementary Figure S6A]. In contrast to ILT-2, pre-incubation with sHLA-G1 did not influence the frequency of the IC molecules CTLA-4, PD-1, TIM-3, or CD95, neither in CD4^+^ nor CD8^+^ T cell subpopulations (Supplementary Figures S6B–E).

### Priming With sHLA-G1 Modulates the Expression of Immune Checkpoint Molecules Exclusively on ILT-2 Positive CD8^+^ T Cells

**FIGURE 1:**
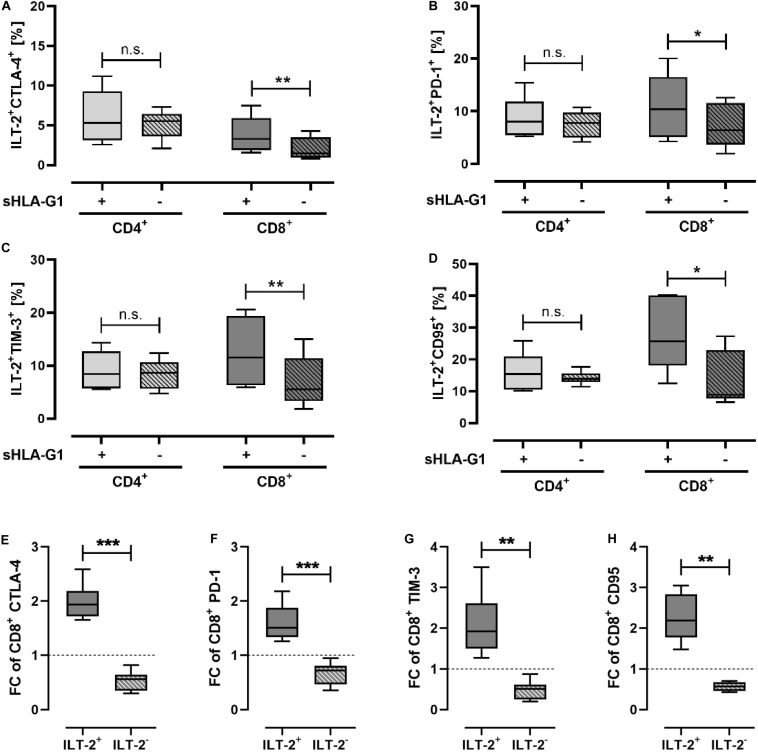
Priming with sHLA-G1 significantly increases surface frequency of immune-modulatory molecules of ILT-2 positive CD8^+^ T cells, but not that of CD4^+^ T cells. Flow cytometric analysis of **(A–D)** ILT-2 positive CD4^+^ and CD8^+^ T cell populations and comparison of **(E–H)** ILT-2 positive and negative CD8^+^ T cells regarding the immune checkpoint molecules CTLA-4, PD-1, TIM-3, and CD95. PBMC of six healthy donors were primed with (+) or without (-) sHLA-G1 overnight followed by stimulation with anti-CD3/CD28 beads for 48 h. **(A–D)** Population frequencies of the CD4^+^ or CD8^+^ ILT-2 positive parent population are given. **(E–H)** For comparison of ILT2 positive and negative CD8^+^ subpopulation, frequencies obtained after stimulation of sHLA-G-primed cells were normalized to the corresponding stimulation obtained without priming and expressed as fold change (FC). Data is presented as median with the 10th and 90th percentile. Statistical significance was determined by two-tailed paired *t*-test. *p ≤ 0.05, **p ≤ 0.01, ***p ≤ 0.001.

As the immunomodulatory effect of sHLA-G1 is preferentially mediated via its interaction with ILT-2, CD4^+^ and CD8^+^ T cells were stratified according to their ILT-2 expression. Focusing on the ILT-2 positive CD4^+^ subpopulation, frequencies of IC molecules were not significantly altered by priming with sHLA-G1. However, among the ILT-2 positive CD8^+^ T cells, priming with sHLA-G1 resulted in a significant increase of CTLA-4, PD-1, TIM-3, and CD95 (Figures 1A–D) frequencies. For the comparison of ILT-2 positive and negative CD8 subpopulations, frequencies of a certain cell population obtained after stimulation of sHLA-G1-primed cells were normalized to the corresponding ones obtained without priming. Strikingly, analysis revealed that the FC of CTLA-4, PD-1, TIM-3, and CD95 (Figures 1E–H) was significantly elevated on ILT-2 positive CD8^+^ T cells compared to ILT-2 negative ones. Comparison of ILT-2 negative and positive CD4^+^ T cells showed no statistically different FC of any IC molecules. Combined these data evidence that sHLA-G -priming mediates an increase in IC molecules specifically on ILT-2 positive CD8^+^ T cells, but not on ILT-2 negative ones.

### Priming With EV Preparations Modulates Immune Checkpoint Molecules Exclusively on ILT-2 Negative CD8^+^ T Cells

**FIGURE 2:**
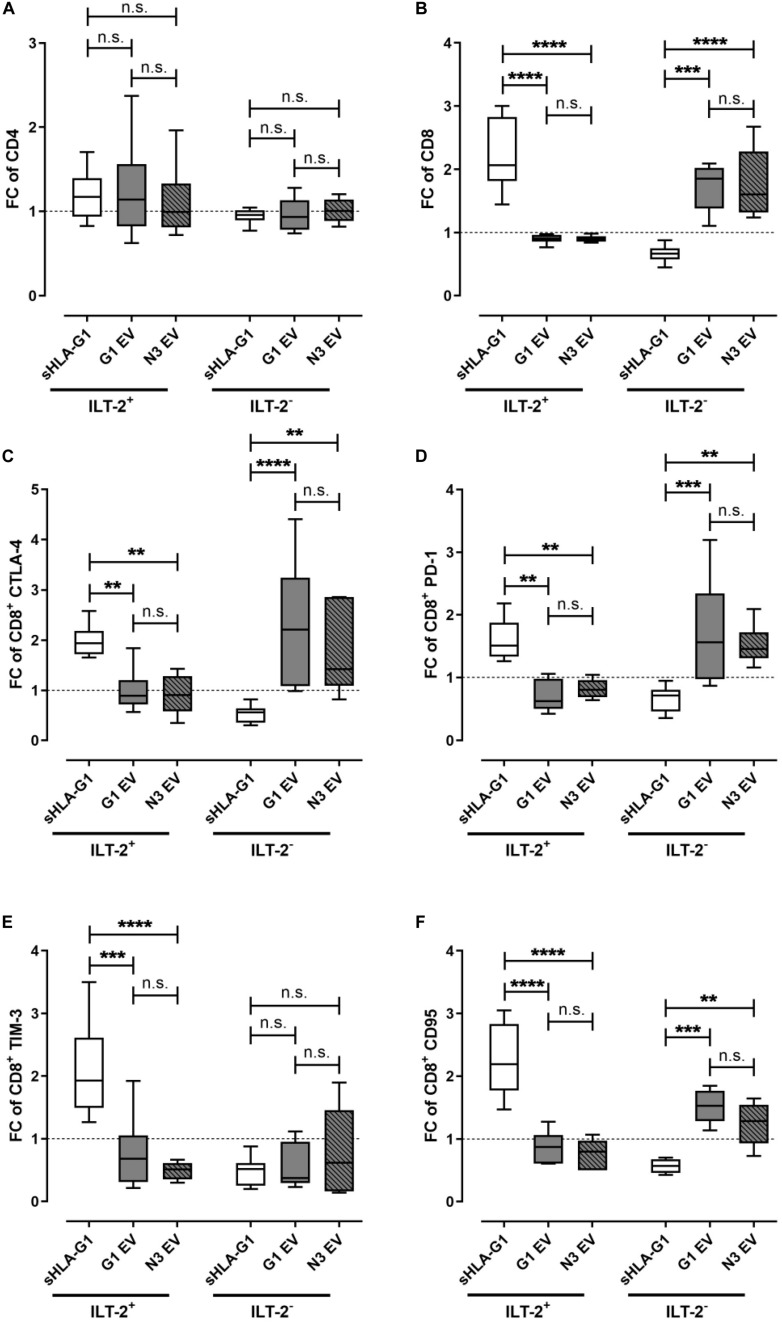
Priming with EV preparations derived from SUM149 cells significantly increase immune-modulatory molecules on ILT-2 negative CD8^+^ T cells compared to sHLA-G-priming. Flow cytometric analysis of **(A)** CD4^+^ and **(B–F)** CD8^+^ T cell populations regarding **(A,B)** ILT-2 and the immune checkpoint molecules **(C)** CTLA-4, **(D)** PD-1, **(E)** TIM-3, and **(F)** CD95. PBMC of six healthy donors were primed with either sHLA-G1, or EV derived from SUM149 LV2 G1-GFP cells (G1 EV) or SUM149 LV2 N3-GFP (N3 EV) overnight followed by stimulation with anti-CD3/CD28 beads for 48 h. For comparison of ILT2 positive and negative CD8^+^ subpopulation, frequencies obtained after stimulation of sHLA-G-primed cells were normalized to the corresponding stimulation obtained without priming and expressed as fold change (FC). Data is presented as median with the 10th and 90th percentile. Statistical significance was determined by two-way ANOVA. **p ≤ 0.01, ***p ≤ 0.001, ****p < 0.0001.

To elucidate the immune-modulatory effect of the different EV preparations compared to sHLA-G1, PBMC were primed either with sHLA-G1 protein or with 40 μg of G1 EV, which corresponded to a mount of 1.2 ng HLA-G1 or with 40 μg N3 EV prior to CD3/CD28 stimulation. For comparison, frequencies of a certain cell population obtained after stimulation of sHLA-G1- or EV-primed cells were normalized to the corresponding ones obtained without priming. Priming with sHLA-G1 or EV did not significantly result in an altered FC of ILT-2 positive or negative CD4^+^ T cells (Figure 2A). However, compared to sHLA-G1-treated cells, EV-priming lead to a significantly reduced FC of ILT-2 positive CD8^+^ T cells, while the FC of its negative counterpart increased significantly (Figure 2B). Concerning the IC molecules CTLA-4, PD-1, and CD95, sHLA-G1- and EV-priming showed opposing effects: among the ILT-2 positive cells, EV-treatment mediated a decline of the FC of CTLA-4^+^, PD-1^+^ and CD95^+^ CD8^+^ T cells compared to sHLA-G1, while among the ILT-2 negative cells, priming with EV resulted in an enhanced FC of CTLA-4^+^, PD-1^+^ and CD95^+^ CD8^+^ T cells compared to sHLA-G1 (Figures 2C,D,F). Further, although not reaching significance, priming with G1 EV induced a substantially elevated (*p* = 0.07) FC of CTLA-4 in ILT-2 negative CD8^+^ T cells compared to N3 EV-primed cells. Considering TIM-3, FC was significantly increased among the ILT-2 positive CD8^+^ T cells upon priming with sHLA-G1 compared to EV-treatment, while among the ILT-2 negative CD8^+^ T cells FC of TIM-3 was not differentially altered by priming with sHLA-G1 or EV preparations (Figure 2E).

### Priming With sHLA-G1 or EV Preparations Drives ILT-2 Positive or Negative CD8^+^ T Cells, Respectively, Toward an Immunosuppressive/Exhausted Phenotype

**FIGURE 3:**
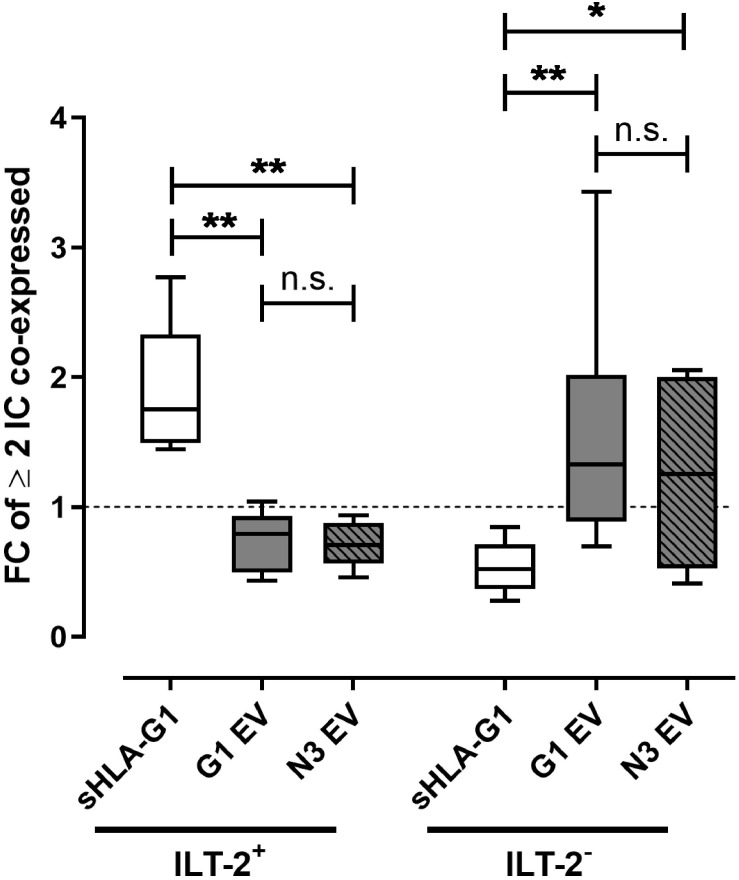
Priming with sHLA-G1 or EV preparations drives ILT-2 positive or negative CD8^+^ T cells, respectively, toward an immunosuppressive/exhausted phenotype. Flow cytometric analysis of the ILT-2 positive and negative CD8^+^ T cell populations regarding the multi-positivity of immune checkpoint molecules (IC) including CTLA-4, PD-1, TIM-3, and CD95. IC greater or equal two was considered as multiple-positivity. PBMC of six healthy donors were primed with or without sHLA-G1 or EV derived from SUM149 LV2 G1-GFP cells (G1 EV) or SUM149 LV2 N3-GFP (N3 EV) overnight followed by stimulation with anti-CD3/CD28 beads for 48 h. For comparison of ILT-2 positive and negative CD8^+^ subpopulation, frequencies obtained after stimulation of sHLA-G-primed cells were normalized to the corresponding stimulation obtained without priming and expressed as fold change (FC). Data is presented as median with the 10th and 90th percentile. Statistical significance was determined by two-tailed paired *t*-test. *p ≤ 0.05, **p ≤ 0.01.

As co-expression of multiple IC molecules is a feature of an immunosuppressive/exhausted phenotype, we analyzed the influence of sHLA-G- or EV-priming on the co-expression of CTLA-4, PD-1, TIM-3, and CD95 on ILT-2 positive and negative CD8^+^ T cells (Figure 3). Strikingly, FC of at least two co-expressed IC was significantly increased upon sHLA-G1-priming compared to EV treatment in ILT-2 positive CD8^+^ T cells, while among the ILT-2 negative CD8^+^ T cells EV-priming led to significantly elevated FC of at least two co-expressed ICs compared to sHLA-G1-priming. Thus, sHLA-G1-priming and priming with EV originated from HLA-G1 positive or negative SUM149 cells appear to act complementary toward an immunosuppressive/exhausted phenotype by targeting either ILT-2 positive or ILT-negative CD8^+^ T cell subpopulations, respectively.

## Discussion

Immune effector cell dysfunction in the periphery of cancer patients can tremendously shape the evolution of tumors by mediating a suppressive/tolerogenic immune microenvironment impeding successful tumor elimination. Both, the expression of IC molecules on peripheral immune cells and soluble forms of HLA-G in the blood are associated with tumor immune escape and consequently discussed as clinical biomarker for disease status and outcome of cancer patients (19). Considering that HLA-G can be secreted as free sHLA-G molecules or via EV, we investigated the contribution of these two forms to the expression of the checkpoint molecules PD-1, CTLA-4, TIM-3, and CD95. In our experimental design we primed PBMC with purified sHLA-G1 protein or with EV preparations derived from the BC cell line SUM149 either HLA-G1 transfected or not prior to T cell stimulation with anti-CD3/CD28 to mimic the situation in peripheral blood. The results of our study demonstrate that priming with purified sHLA-G1 protein before T cell activation resulted (i) in enhanced frequencies of ILT-2 positive CD8^+^ T cells, and (ii) in enhanced frequencies of the IC molecules CTLA-4, PD-1, TIM-3, and CD95 exclusively on ILT-2 positive CD8^+^ T cells. (iii) Priming with HLA-G1 positive or negative EV preparations prior to T cell activation lead to enhanced frequencies of CTLA-4, PD-1, and CD95 exclusively on ILT-2 negative CD8^+^ T cells. (iv) Accordingly, the co-expression of at least two IC, being indicative for a pronounced immunosuppressive or exhausted phenotype, was enhanced on ILT-2 positive CD8^+^ T cells upon sHLA-G1 priming and on ILT-2 negative CD8^+^ T cells upon EV priming. (v) Combined, priming with sHLA-G1 and EV derived from HLA-G1 positive or negative transfected SUM149 BC cells seem to affect CD8^+^ T cells complementary by targeting either the ILT-2 positive or ILT-2 negative subpopulation, respectively.

We demonstrated that priming with sHLA-G1 significantly increased the frequency of ILT-2 on CD8^+^ T cells, while frequencies of classical immune-modulatory molecules such as CTLA-4, PD-1, TIM-3, and CD95 were not altered. In fact, it has already been demonstrated that HLA-G1 is capable of signaling transcriptional and phenotypical changes in immune cells as described by the upregulation of ILT-2, ILT-3, ILT-4, and KIR2DL4 in antigen presenting cells, NK cells, and T cells (37). ILT-2 expression is considered to be more prominent on CD8^+^ T cells compared to CD4^+^ T cells with almost exclusive presence on previously activated cells (38–40). Our data, however, showed similar frequencies of ILT-2 in CD3/CD28 stimulated unprimed CD4^+^ and CD8^+^ T cells, whereas ILT-2 frequencies were more pronounced within the CD8^+^ subpopulation upon sHLA-G1-priming.

Although generic analysis of CD4^+^ and CD8^+^ T cells did not reveal any sHLA-G-mediated alteration of tolerogenic molecules despite ILT-2, stratification of CD4^+^ and CD8^+^ T cells into ILT-2 positive and negative subpopulations revealed that sHLA-G1 predominantly and preferentially influences the IC molecule profile on ILT-2 positive CD8^+^ T cells as compared to their ILT-2 negative counterpart. This is in line with Jacquier et al. (23) who requested that functional analyses of the immunosuppressive potential of HLA-G on PBMC should be refined toward ILT-2 positive, and thus, HLA-G-sensitive, cells.

A major mechanism by which tumor cells can impair immune effector function is hijacking of ICs as that mediated by the PD-1/PD-L1 pathway (41). Probably, different ICs (e.g., PD-1, CTLA-4) and HLA-G influence each other as it has been reported, for instance, for TIM-3 expression on CD8^+^ tumor infiltrating lymphocytes that is closely associated with PD-1 expression (42). Indeed, this is the first study describing a synergy of sHLA-G1 and the frequency of IC molecules on certain T cell subsets. In this context, Contini et al. (18) have already reported that sHLA-G can bind CD8 without T cell receptor interaction inducing apoptosis in activated CD8^+^ T cells through upregulation of FasL expression. Further, up-regulation of the expression of cell surface molecules such as FasL in cancer cells may mediate the dampening of cytotoxic T cell attacks (41). Thus, upregulation of the corresponding receptor CD95 (Fas) on sHLA-G1-primed CD8^+^ T cells – as observed in our study – may increase the probability of apoptotic T cell death as both, the ligand and the corresponding receptor are upregulated. Similarly, cancer cells express high levels of inhibitory ligands such as PD-L1 and PD-L2, which, upon binding to PD-1 on T cells inhibits response of T cells toward cancer cells (41). Again, sHLA-G1 priming resulted in elevated frequency of PD-1 on CD8^+^ T cells, potentially contributing to the establishment of immune escape via the PD-1/PD-L1 axis. Hence, sHLA-G1-priming reinforces an immunosuppressive TME rendering ILT-2 positive cytotoxic T cells unresponsive to cancer cells. Similarly, Dumont et al. (43) have demonstrated that CD8^+^ tumor infiltrating lymphocytes (TIL) expressing ILT-2 showed a higher cytotoxicity and IFNγ production compared to their ILT-2 negative or PD-1 expressing counterparts and that cytotoxicity of ILT-2 positive TIL, but not that of ILT-2 negative or PD-1 positive TIL could be inhibited by HLA-G. Combined, Dumonts study and our study suggests that various IC pathway act concomitantly in the TME.

Hitherto, the majority of clinical studies analyzed sHLA-G molecules as a prognostic marker in various malignancies (19). Previously, we established that discrimination of sHLA-G forms represents diametric prognostic impacts on the clinical outcome of BC patients (21) and that only HLA-G_EV_, but not the total amount of sHLA-G is an independent predictor for progression in ovarian cancer patients (20). Thus, we compared the effect of sHLA-G1 with that of EV derived from CM of SUM149 cells, transfected with (G1 EV) or without (N3 EV) HLA-G1. In our study we demonstrated that (iv) sHLA-G1 and EV impact CD8^+^ T cells complementary: while sHLA-G predominantly influenced ILT-2 positive cells, ILT-2 negative cells were highly affected by EV. Thereby, the surface expression pattern of immune-modulatory molecules on CD8^+^ T cells was substantially influenced toward an immunosuppressive/exhausted phenotype by both, sHLA-G and HLA-G_EV_ in an ILT-2-dependent or -independent manner, respectively. Of note, ILT-2 positive cells – and thus, per definition HLA-G-sensitive cells – represent only a minority of immune subsets (23). Hence, it is tempting to speculate that HLA-G_EV_ have a larger pool of cells to interact with, potentially explaining the prognostic relevance of HLA-G_EV,_ but not of total sHLA-G, in breast and ovarian cancer patients (20, 21). However, the prognostic potential of sHLA-G or HLA-G_EV_ might be changed in situations with increased frequencies of ILT-2 positive CD8^+^ T cells such as during aging or chronic viral infections. Additionally, we demonstrated that our EV preparations carry classical HLA class I molecules. As classical HLA class I molecules are generally not expressed as a dimer, it is unlikely that they interact with the ILT-2 receptor which preferentially binds HLA-G dimers (44) rationalizing sHLA-G’s preference to bind to the ILT-2 receptor on T cells. On the other hand, CD8 is the cognate receptor of classical HLA class I molecules explaining the preference of EV to interact with ILT-2 negative CD8^+^ T cells.

Moreover, these results raise questions concerning the relation between these HLA-G structures in physiological and pathological situations. How does priming with a combination of sHLA-G1/HLA-G_EV_ affect the phenotype of T cells? Is the effect of sHLA-G1 and HLA-G_EV_ in the periphery additive, synergistic or competitive? Does one of the structures dominate? What is the ratio of sHLA-G1 to HLA-G_EV_ structures in the periphery of cancer patients in comparison to healthy individuals?

Despite these open questions, our data underline that EV are soluble carriers enhancing the immunosuppressive properties of the TME. As EV represent multifactorial vehicles, it should be acknowledged that the composition of the applied EV preparations is not restricted to HLA-G. In fact, TEV may expose ligands or antigens on their membrane that interact with cellular HLA receptors, thereby altering immune function (45). Moreover, TEV can carry immunosuppressive molecules such as FasL, TGF-β1, TRAIL, PD-L1, and NKG2D ligands, which are involved in immunosuppression (46, 47). Of note, TEV can affect the behavior of immune cells through receptor-ligand binding interaction or by internalization (10). Recently, it has been reported that the modulation of T cell function by TEV is not exerted via internalization by T cells, but rather via signaling molecules that they carry and deliver to the cell surface (46). Accordingly, we demonstrated that EV – irrespective of their composition – modify ILT-2 negative cells, while ILT-2 positive cells are unaffected. Notably, EV preparations are a heterogeneous group of diverse EV subsets. Comprehensive analysis of the EV preparations, especially considering classical IC molecules, might shed further light on the functionality of the EV-driven immunological modifications. In this context, elucidating the structural diversity of HLA-G on EV with regards to the monomeric vs. dimeric conformation, may explain the affinity toward ILT-2 negative CD8^+^ T cells observed under our experimental conditions. Of note, another open, but highly interesting question is the sensitivity of CD8^+^ T cells toward priming followed by anti-CD3/CD28 stimulation. Two major mechanisms by which TEV can contribute to tumor evasion are the initiation of apoptosis in cytotoxic CD8^+^ T cells and the conversion of conventional CD4^+^ T cells into regulatory T cells (48). Thus, the sensitivity of CD8^+^ T cells to priming with sHLA-G forms observed in our study might be explained by our lack of emphasis on the regulatory phenotype of CD4^+^ T cells biasing the analyses toward the CD8^+^ T cell subpopulation.

A limitation of our study is the lack of blocking experiments demonstrating HLA-G specificity of the G1 EV preparation and the lack of functional assays demonstrating the functionality of T cells with an immunosuppressive phenotype. Generally, the capability of G1 EV and N3 EV to modify the surface expression of immune-modulatory molecules was similar. Nevertheless, our data clearly show an EV-driven effect compared to sHLA-G-priming or compared to T cell stimulation without priming. Moreover, FC of CD95^+^ CD8^+^ T cells was tentatively increased upon priming with G1 EV compared to N3 EV. Here, homogeneous HLA-G_EV_ preparations might enhance the effects observed in our study; however, due to the current technical limitations in the EV field, purification of homogeneous EV fractions is impossible.

Concluding, our data elucidate that priming of immune effector cells by discrete sHLA-G forms, including purified sHLA-G1 protein as well as HLA-G1 positive and negative EV, differentially modifies the phenotype of these cells. Here, we report that sHLA-G1 preferentially influences ILT-2 positive CD8^+^ T cells, while HLA-G_EV_ mediate phenotypic alterations in ILT-2 negative CD8^+^ T cells. Thus, it seems that discrete soluble HLA-G structures affect ILT-2 positive and ILT-2 negative CD8^+^ T cells complementary suggesting that HLA-G-mediated inhibition of effector immune cells is not restricted to cells expressing the corresponding receptor ILT-2. Further, we provide first evidence that immune-modulation by soluble HLA-G might involve other IC molecules toward an immunosuppressive or exhausted phenotype. Combined, our data highlight that analyses of HLA-G functionality should be extended to discrete structures reinforcing its complexity in the periphery of cancer patients.

## Data Availability Statement

The raw data supporting the conclusions of this article will be made available by the authors, without undue reservation.

## Author Contributions

ES: study design, data acquisition, statistical analysis, and manuscript writing. JL: provision of transfected SUM149 cells and manuscript editing. G-GH and CB-D: characterization and provision of sHLA-G1 protein and manuscript editing. EC and PH: manuscript editing. VR: study design, statistical analysis, and manuscript writing. All authors contributed to the article and approved the submitted version.

## Conflict of Interest

VR declares research support and travel support from Bristol Myers Squibb. The remaining authors declare that the research was conducted in the absence of any commercial or financial relationships that could be construed as a potential conflict of interest.

## Supplementary Material

FIGURE S1 EV characterization by western blotting. Marker expression analysis for **(A)** Calnexin, Tsg101, classical HLA class I, Syntenin, and CD81, and **(B)** HLA-G in EV fractions derived from SUM149 cell lines either transfected with a control vector (N3) or with HLA-G (G1) and their respective cell lysates. Cell lysates were used as positive control for Calnexin. Cell culture supernatants were collected and EV were purified by Tangential Flow Filtration and Ultra-centrifugation.

FIGURE S2 Stimulation of PBMC with anti-CD3/CD28 beads increases frequency of surface expression of several markers on CD4^+^ and CD8^+^ T cells. Flow cytometric analysis of CD4^+^ and CD8^+^ T cell populations regarding **(A)** the HLA-G receptor ILT-2, and the immune checkpoint molecules **(B)** CTLA-4, **(C)** PD-1, **(D)** TIM-3, and **(E)** CD95. PBMC of six healthy donors were stimulated with (+) or without (-) anti-CD3/CD28 beads for 48 h. Population frequencies of the CD4^+^ or CD8^+^ parent population are given. Data is presented as median with the 10th and 90th percentile. Statistical significance was determined by two-tailed paired *t*-test. ^∗^*p* ≤ 0.05, ^∗∗^*p* ≤ 0.01.

FIGURE S3 Effects of pre-incubation of PBMC on the viability of CD4^+^ and CD8^+^ T cells. Flow cytometric analysis of T cell populations regarding **(A)** the viability of CD4^+^ and CD8^+^ T cells and **(B)** the expression of CD95 on dead CD4^+^ and CD8^+^ T cells and **(C)** the expression of CD95 on dead ILT-2 positive and negative CD8^+^ T cells. PBMC of six healthy donors were pre-incubated **(A,B)** with (+) or without (-) sHLA-G1, or **(C)** with sHLA-G1, G1 EV or N3 EV prior to stimulation with anti-CD3/CD28 beads for 48 h. **(A,B)** Population frequencies of the CD4^+^ or CD8^+^ parent population are given. **(C)** Data was normalized to stimulation without pre-incubation and is given as fold change. Data is presented as median with the 10th and 90th percentile. Statistical significance was determined by **(A,B)** two-tailed paired *t*-test (^∗^*p* ≤ 0.05) or **(C)** two-way ANOVA (^∗^*p* ≤ 0.05, ^∗∗^*p* ≤ 0.01, ^∗∗∗^*p* ≤ 0.001).

FIGURE S4 General gating strategy of flow cytometric analysis to characterize T cell subpopulations in PBMC. Total lymphocytes were first gated on forward scatter (FSC)/side scatter (SSC) plot. After gating on single cells, dead cells were dismissed via the fluorescent dye Live/Dead^TM^. T cells were identified by the expression of the T cell receptor CD3. T cells were classified as CD8^+^ (CD3^+^CD8^+^) or CD4^+^ (CD3^+^CD8^–^) T cells. **(A)** Within the CD4^+^ and CD8^+^ population expression frequencies of ILT-2, CTLA-4, PD-1, TIM-3, and CD95 were determined. **(B)** CD4^+^ and CD8^+^ population were distinguished by ILT-2. Within the ILT-2^+^ and ILT-2^–^ CD4^+^ or CD8^+^ T cell populations expression frequencies of CTLA-4, PD-1, TIM-3, and CD95 were assessed. Data were analyzed using the Kaluza software and population frequencies expressed as percent of the CD4^+^ and CD8^+^ parent population or the CD4^+^ or CD8^+^ and ILT-2^+^ or ILT-2^–^ parent population.

FIGURE S5 General analysis strategy of multi-positive T cells. A tree analysis including gates of ILT-2, PD-1, CTLA-4, TIM-3, and CD95 was performed based on the CD4^+^ or CD8^+^ T cell population divided into ILT-2 positive and negative subpopulation resulting in 32 receptor combinations (16 for ILT-2 positive and ILT-2 negative CD4^+^/CD8^+^ T cells, respectively). Due to low numbers of recorded frequencies for multi-positive cells, frequencies of cells with more than 1 receptor were added up for further analysis. A representative analysis of the CD8^+^ population is shown.

FIGURE S6 Priming with sHLA-G1 significantly increases frequency of ILT-2 on CD8^+^ T cells, while frequency of immune checkpoint molecule is not altered by priming with sHLA-G1. Flow cytometric analysis of CD4^+^ and CD8^+^ T cell populations regarding **(A)** the HLA-G receptor ILT-2, and the immune checkpoint molecules **(B)** CTLA-4, **(C)** PD-1, **(D)** TIM-3, and **(E)** CD95. PBMC of six healthy donors were primed with (+) or without (-) sHLA-G1 overnight followed by stimulation with anti-CD3/CD28 beads for 48 h. Population frequencies of the CD4^+^ or CD8^+^ parent population are given. Data is presented as median with the 10th and 90th percentile. Statistical significance was determined by two-tailed paired *t*-test. ^∗^*p* ≤ 0.05, ^∗∗^*p* ≤ 0.01.

TABLE S1 EV characterization by Nanoparticle Tracking Analysis and protein assay. Particle concentration and particle size of EV fractions derived from SUM149 cell lines either transfected with a control vector (N3) or with HLA-G (G1) was determined by Nanoparticle Tracking Analysis, while total protein concentration was assessed by MacroBCA. Cell culture supernatants were collected and EV were enriched by Tangential Flow Filtration and Ultra-centrifugation.
