# Supplementary figures and images for: Soluble HLA-G and HLA-G Bearing Extracellular Vesicles Affect ILT-2 Positive and ILT-2 Negative CD8 T Cells Complementary
Source: Front Immunol. 2020 Aug 21;11:2046. doi: 10.3389/fimmu.2020.02046 (PMC7472666; doi:10.3389/fimmu.2020.02046)

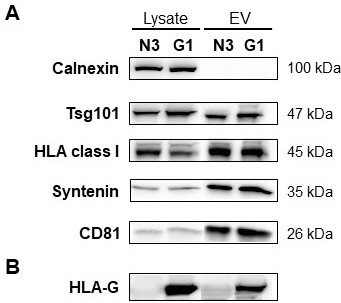

Supplement: FIGURE S1 — EV characterization by western blotting. Marker expression analysis for (A) Calnexin, Tsg101, classical HLA class I, Syntenin, and CD81, and (B) HLA-G in EV fractions derived from SUM149 cell lines either transfected with a control vector (N3) or with HLA-G (G1) and their respective cell lysates. Cell lysates were used as positive control for Calnexin. Cell culture supernatants were collected and EV were purified by Tangential Flow Filtration and Ultra-centrifugation. [file Image_1.jpg]

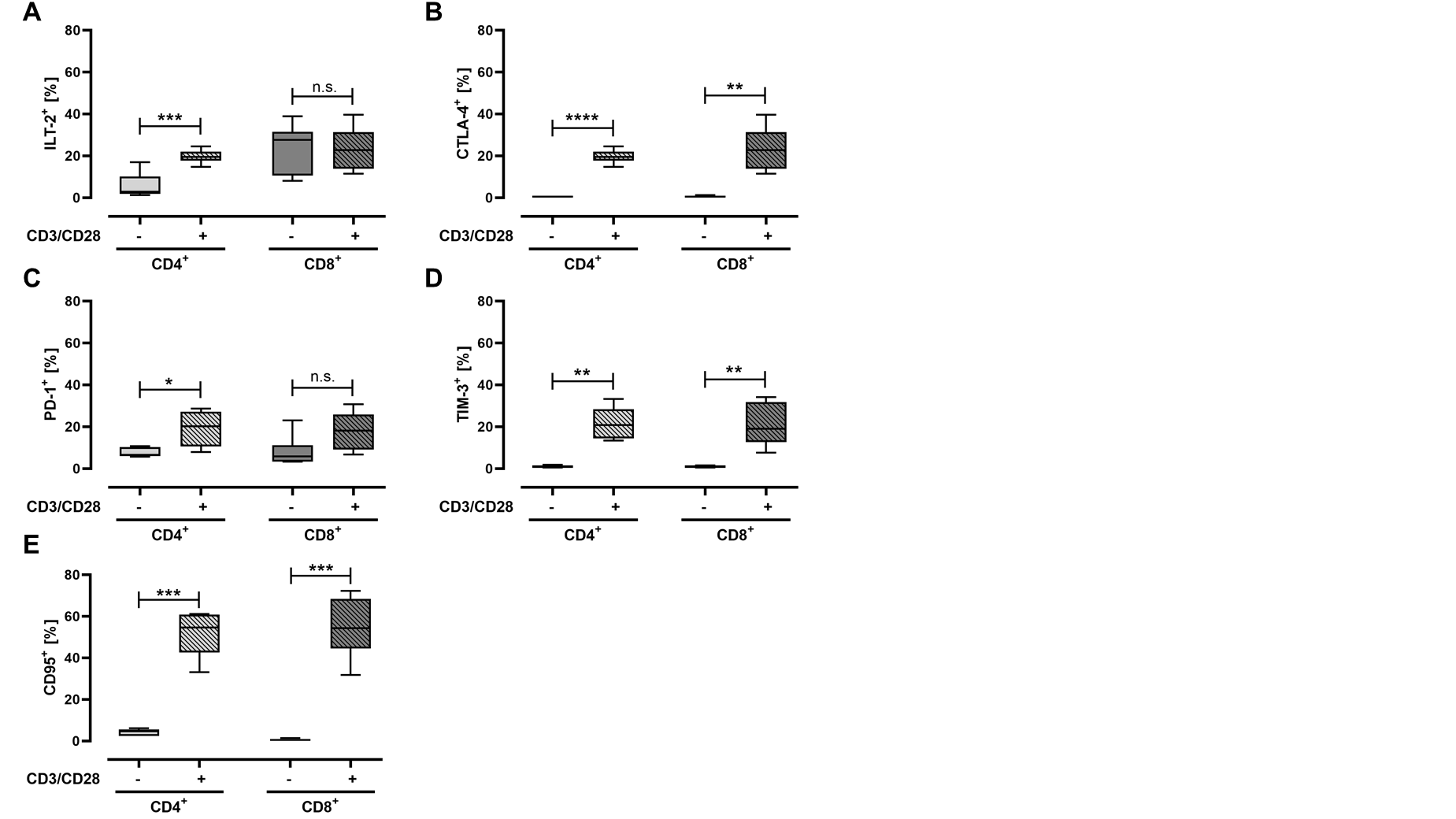

Supplement: FIGURE S2 — Stimulation of PBMC with anti-CD3/CD28 beads increases frequency of surface expression of several markers on CD4+ and CD8+ T cells. Flow cytometric analysis of CD4+ and CD8+ T cell populations regarding (A) the HLA-G receptor ILT-2, and the immune checkpoint molecules (B) CTLA-4, (C) PD-1, (D) TIM-3, and (E) CD95. PBMC of six healthy donors were stimulated with (+) or without (-) anti-CD3/CD28 beads for 48 h. Population frequencies of the CD4+ or CD8+ parent population are given. Data is presented as median with the 10th and 90th percentile. Statistical significance was determined by two-tailed paired t-test. ∗p ≤ 0.05, ∗∗p ≤ 0.01. [file Image_2.TIF]

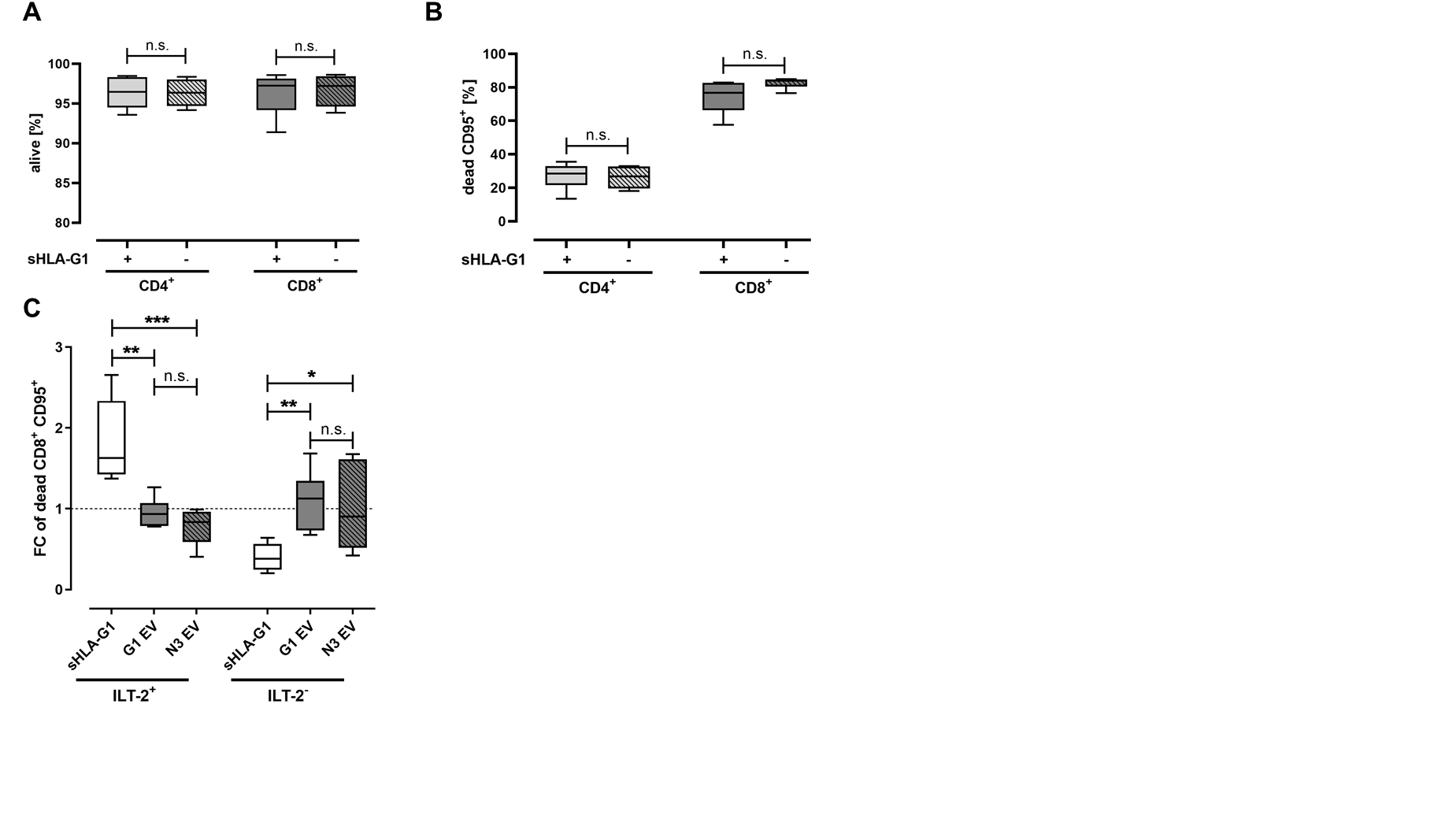

Supplement: FIGURE S3 — Effects of pre-incubation of PBMC on the viability of CD4+ and CD8+ T cells. Flow cytometric analysis of T cell populations regarding (A) the viability of CD4+ and CD8+ T cells and (B) the expression of CD95 on dead CD4+ and CD8+ T cells and (C) the expression of CD95 on dead ILT-2 positive and negative CD8+ T cells. PBMC of six healthy donors were pre-incubated (A,B) with (+) or without (-) sHLA-G1, or (C) with sHLA-G1, G1 EV or N3 EV prior to stimulation with anti-CD3/CD28 beads for 48 h. (A,B) Population frequencies of the CD4+ or CD8+ parent population are given. (C) Data was normalized to stimulation without pre-incubation and is given as fold change. Data is presented as median with the 10th and 90th percentile. Statistical significance was determined by (A,B) two-tailed paired t-test (∗p ≤ 0.05) or (C) two-way ANOVA (∗p ≤ 0.05, ∗∗p ≤ 0.01, ∗∗∗p ≤ 0.001). [file Image_3.TIF]

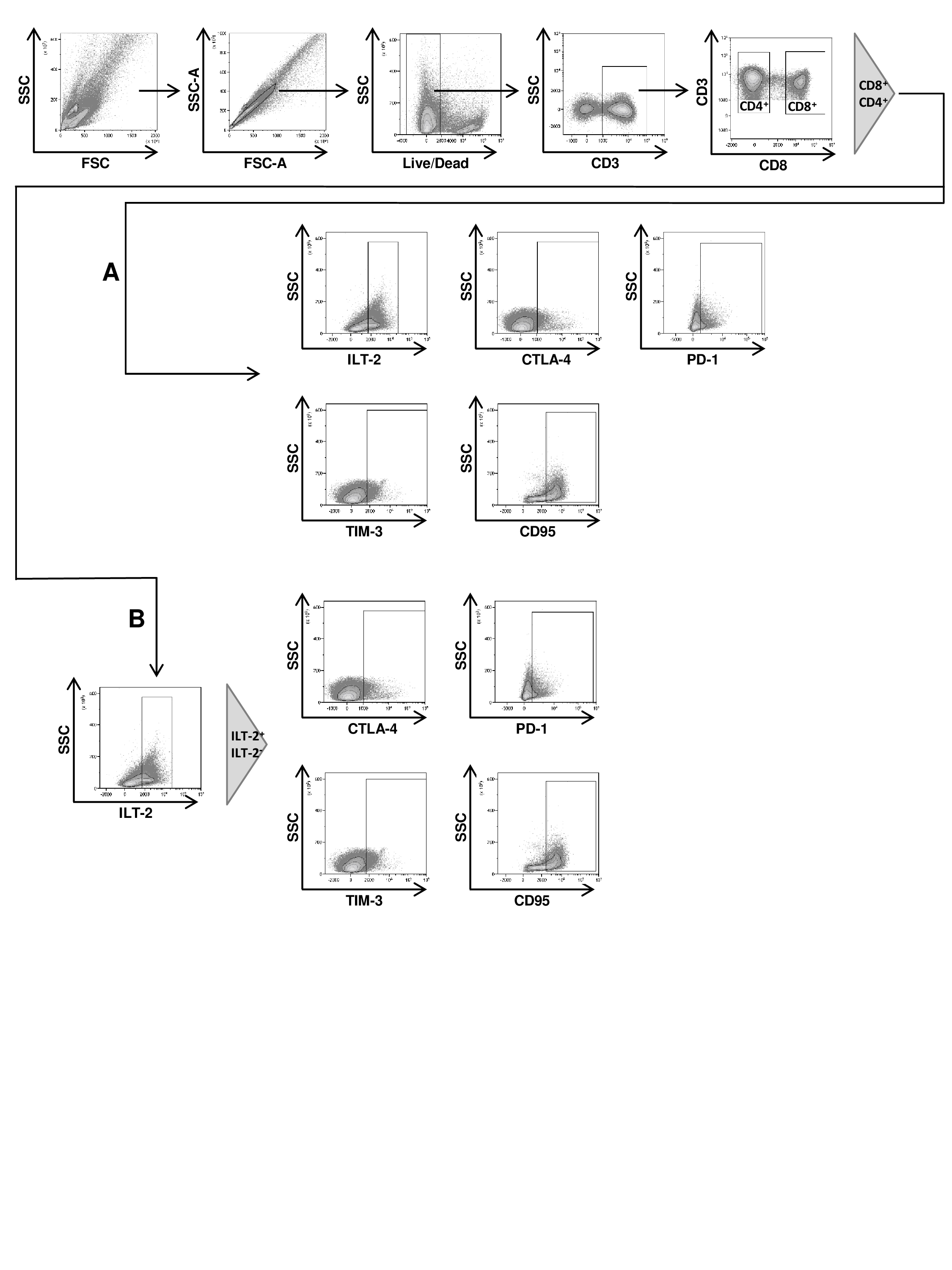

Supplement: FIGURE S4 — General gating strategy of flow cytometric analysis to characterize T cell subpopulations in PBMC. Total lymphocytes were first gated on forward scatter (FSC)/side scatter (SSC) plot. After gating on single cells, dead cells were dismissed via the fluorescent dye Live/DeadTM. T cells were identified by the expression of the T cell receptor CD3. T cells were classified as CD8+ (CD3+CD8+) or CD4+ (CD3+CD8–) T cells. (A) Within the CD4+ and CD8+ population expression frequencies of ILT-2, CTLA-4, PD-1, TIM-3, and CD95 were determined. (B) CD4+ and CD8+ population were distinguished by ILT-2. Within the ILT-2+ and ILT-2– CD4+ or CD8+ T cell populations expression frequencies of CTLA-4, PD-1, TIM-3, and CD95 were assessed. Data were analyzed using the Kaluza software and population frequencies expressed as percent of the CD4+ and CD8+ parent population or the CD4+ or CD8+ and ILT-2+ or ILT-2– parent population. [file Image_4.TIFF]

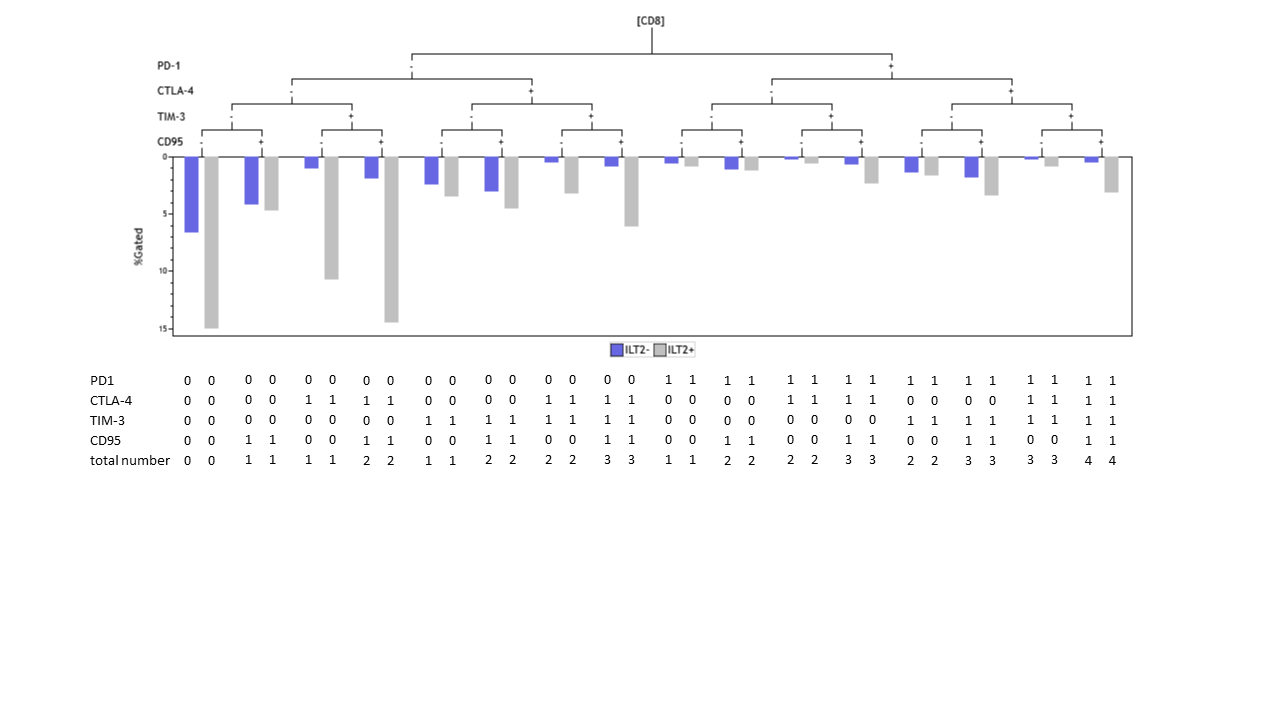

Supplement: FIGURE S5 — General analysis strategy of multi-positive T cells. A tree analysis including gates of ILT-2, PD-1, CTLA-4, TIM-3, and CD95 was performed based on the CD4+ or CD8+ T cell population divided into ILT-2 positive and negative subpopulation resulting in 32 receptor combinations (16 for ILT-2 positive and ILT-2 negative CD4+/CD8+ T cells, respectively). Due to low numbers of recorded frequencies for multi-positive cells, frequencies of cells with more than 1 receptor were added up for further analysis. A representative analysis of the CD8+ population is shown. [file Image_5.TIF]

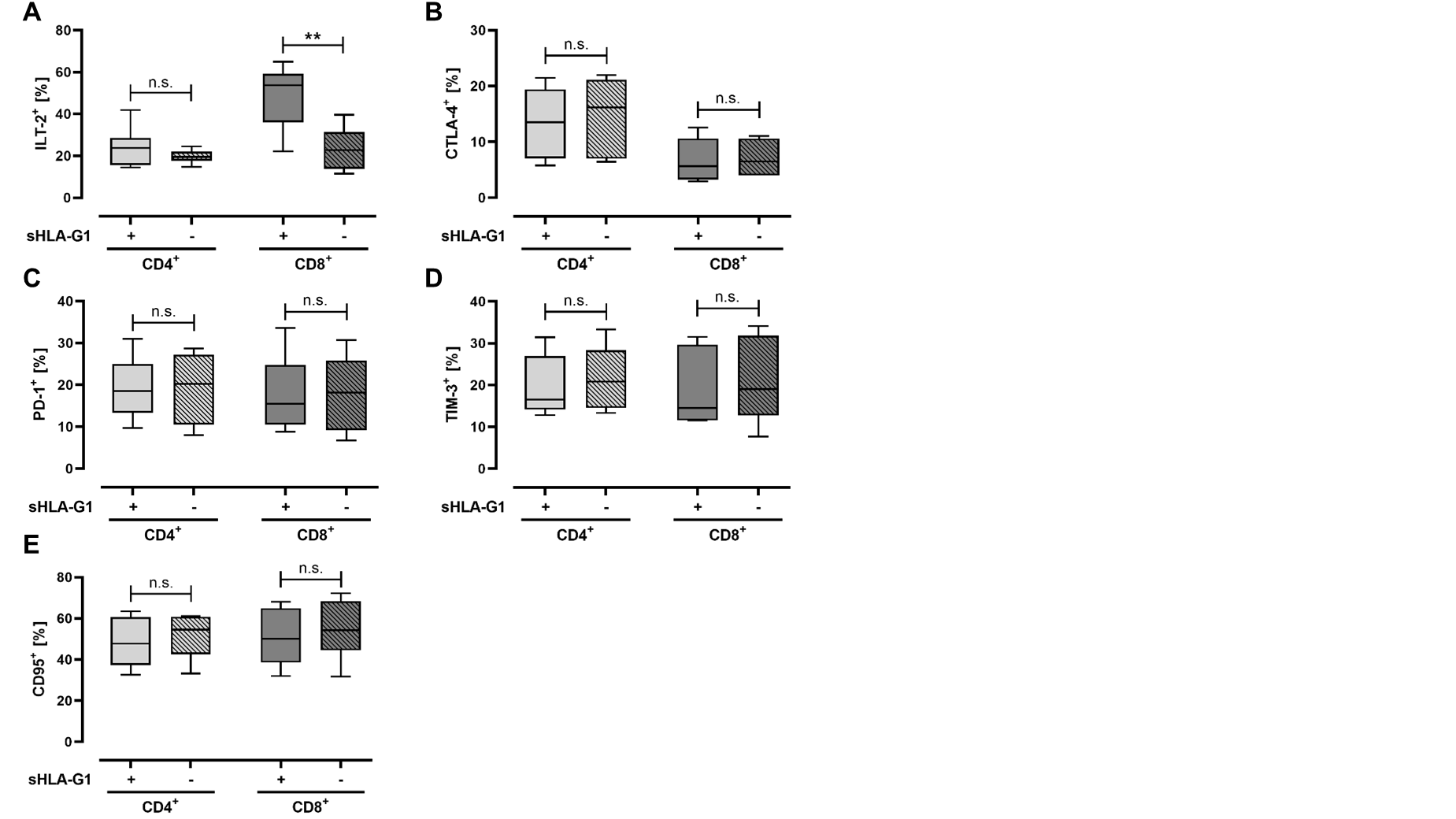

Supplement: FIGURE S6 — Priming with sHLA-G1 significantly increases frequency of ILT-2 on CD8+ T cells, while frequency of immune checkpoint molecule is not altered by priming with sHLA-G1. Flow cytometric analysis of CD4+ and CD8+ T cell populations regarding (A) the HLA-G receptor ILT-2, and the immune checkpoint molecules (B) CTLA-4, (C) PD-1, (D) TIM-3, and (E) CD95. PBMC of six healthy donors were primed with (+) or without (-) sHLA-G1 overnight followed by stimulation with anti-CD3/CD28 beads for 48 h. Population frequencies of the CD4+ or CD8+ parent population are given. Data is presented as median with the 10th and 90th percentile. Statistical significance was determined by two-tailed paired t-test. ∗p ≤ 0.05, ∗∗p ≤ 0.01. [file Image_6.TIF]
