# Supplementary material for: Soluble HLA-G and HLA-G Bearing Extracellular Vesicles Affect ILT-2 Positive and ILT-2 Negative CD8 T Cells Complementary
Source: Front Immunol. 2020 Aug 21;11:2046. doi: 10.3389/fimmu.2020.02046 (PMC7472666; doi:10.3389/fimmu.2020.02046)
Supplement: TABLE S1 — EV characterization by Nanoparticle Tracking Analysis and protein assay. Particle concentration and particle size of EV fractions derived from SUM149 cell lines either transfected with a control vector (N3) or with HLA-G (G1) was determined by Nanoparticle Tracking Analysis, while total protein concentration was assessed by MacroBCA. Cell culture supernatants were collected and EV were enriched by Tangential Flow Filtration and Ultra-centrifugation. [file Table_1.DOCX]

|  | **Particle number^1^ [particle/mL]** | **Particle size^1^ [nm]** | **Total protein concentration^2^ [ng/µL]** |
| --- | --- | --- | --- |
| **SUM149 G1 EV** | 1.8 x10e11 | 136.7 | 3700 |
| **SUM149 N3 EV** | 3.9 x10e11 | 133.3 | 4800 |

^1^determined by Nanoparticle Tracking Analysis (Particle Metrix, Meerbusch, Germany)

^2^determined by MacroBCA (Thermo Scientific, Darmstadt, Germany)
